# Supplementary material for: Multi- class classification of breast cancer abnormalities using Deep Convolutional Neural Network (CNN)
Source: PLoS One. 2021 Aug 26;16(8):e0256500. doi: 10.1371/journal.pone.0256500 (PMC8389446; doi:10.1371/journal.pone.0256500)
Supplement: Attachment — Submitted filename: Response to Reviewers.docx [file pone.0256500.s001.docx]

**Response to Editor**

Dear Editor,

First of all, I would like to thank you for the reviews. The comments/ suggestions from the reviewers were valuable and has allowed us to improve our paper. I have put all the changes/ details in a table as below:

| **Additional Requirements** | |
| --- | --- |
| 1. Please ensure that your manuscript meets PLOS ONE's style requirements, including those for file naming. The PLOS ONE style templates can be found at  https://journals.plos.org/plosone/s/file?id=wjVg/PLOSOne_formatting_sample_main_body.pdf and  https://journals.plos.org/plosone/s/file?id=ba62/PLOSOne_formatting_sample_title_authors_affiliations.pdf | Have revised the manuscript accordingly |
| Please amend your Methods section to provide links to all of the online data sets used in the study | Have provided links of the datasets |
| **Reviewer 1** | |
| Introduction part is well written. Introduction/Related work must conclude with clear research gap. | The research gap is the lack of research in the classification of breast cancer abnormalities. The last line of the Related Work has been revised to stress on the research Gap |
| Data set description is clearly mentioned in the proposed work. Authors have used CLAHE for image pre-processing. Need to analyze this image pre-processing technique on various parameters, namely, Illumination corrections, Blur and focus corrections, Filtering and noise removal, Thresholding Edge enhancements, Morphology, Segmentation, Region processing and filters, Point processing, Color space conversions (Whichever is appropriate for given data set). | In this work, images are pre-processed. First, the various filters applied to enhance the image and to remove possible noises in the image are analysed. These filters are Wiener filter and Median filters. The "Data Pre-processing" techniques has discussed the Wiener filter and Median filters and have critically given its analysis. Eventually CLAHE was chosen. Some part of the sentences in this section have been re-worded to highlight the comment. |
| Results section need to be improvised. Authors may implement classification using other deep learning models and compare results in terms of validation accuracy and loss, with enhanced CNN model. | Other deep learning model (ResNet50) was implemented and results were discussed. This part is reflected in " **Transfer Learning Using Pre-trained ResNet50".** The results are provided are in **the Results and Performance section.** Furthermore, an enhanced model was developed where all the parameters concerned were experimented with. The results are also compared in the results section. The comparison can be made in terms of testing accuracy. To compare the ResNet50 and our proposed model, a table is added in the**Results and Performance Section** to summarise the results. Note that the validation accuracy is used for the purpose of overfitting. The performance lies in the testing accuracy of the models. |
| Authors are suggested to include more analysis graphs in the results section. Authors may analyze the output by considering various epochs in the training/testing phase. Authors need to mention various parameters used during the implementation of CNN. The typical parameters could be Activation Function, Optimizer, Loss function, Metric (Whichever is suitable for proposed work). | In the result section, one more table has been added to compare the models implemented. One very imprtant feature that has not been considered so far in literature is the learning rate. One main contribution in this work is the learning rate which is explicitly discussed in section **Results and Performance.** This change is reflected in the validation accuracy listed in this section. Thus parameter chosen is the learning rate, which has been discussed and supported by the equations listed. |
| Authors are suggested to include Future work in this area. | This has been added in the conclusion. To improve the diagnosis of breast cancer, more breast image datasets taken under varied conditions should be made available to the research community. Following, image processing and artificial intelligence techniques can be applied to determine the most appropriate imaging technology to be adopted. Alongside, techniques should further be improved to detect and classify the abnormality in the breast at an early stage, thus, determining the most appropriate treatment to be administered to the patient in the hope of reducing deaths caused by breast cancer. |
| **Reviewer 2** | |
| The language expression needs to be more concise. There are many grammatical mistakes in the paper, so I suggest author revise them. | This has been taken care of in the revised version. |
| The Development of the Enhanced CNN Model should not too much describe the previous work, but focus on the work of this paper | Some lines that may be obvious for someone familiar in CNN has been removed in the revised version. Otherwise, those which are important for understand the model are kept in the final manuscript. |
| The Related Work part mentioned that 30% test set and 70% training set were carried out, and after combining CBIS-DDSM Dataset, It is also divided into 60% training set and 40% test set. The Results and Performance Evaluation section also mentions having a validation set, How exactly did you divide the data set, please explain in detail. | This has been addressed in section Results and Performance Evaluation: The training set, which represents the sample data, is used to fit the model. The latter learns from this data, which is 60% taken from the dataset. To ensure that the four classes (mass, carcinoma, asymmetry and calcifications) are well represented, 15% from each category were considered. To avoid unbiassed evaluation on the training set, a validation set is chosen to tune the parameters. Out of the remaining 40%, 20% was allocated to the evaluation set and 20 % to the test set. The main role of the validation set is to choose the best combination of parameters for our model.   Note that we can take 70-30 Or 60-40. We have considered 60-40 since we have used part of the 40(that is 20%) for the validation set. It is very important to fine tune the parameters since the model might be learning extra unimportant features. The abnormalities are very small at times in breast cancer and thus, feature extraction is complex. |
| The format of the table needs to be adjusted so that the words are on the same line as possible for easy reading. | Format of the Table has been adjusted |
| Formula labels are duplicated and there are two different formulas (5).  Please check carefully | The labels of the formula has been revised. |
| The reference is suggested to be in a unified format, and the number of reference 8 is wrong. | Number of reference 8 is modified. The typo (b) should be read as 8. Updates done |
| Please redesign pictures 4, 5 and 6 to ensure the sharpness of the pictures | Please advise if I further need to change the diagrams since it was done earlier whereby the Journal already checked the images. Please let me know if it needs further modification. |
| .Figure 5 should not take a random output as the result, but should look at the overall accuracy and the change of loss. | The overall accuracy and change of loss is being discussed in the paper. Figure 5 might not add any value to the explanation and has been eliminated. |
| Try to keep the form to one page. | It is difficult to fit the whole table in one page. I have repeated the header since it spans on multiple page. |
| The paper should refer to the latest research work. A Review of Deep-Learning-Based Medical Image Segmentation Methods, Sustainability, 2021, 13(3), 1224.  An image enhancement algorithm of video surveillance scene based on deep learning, IET Image Processing, 2021, https://doi.org/10.1049/ipr2.12286 | I have referred the paper: "A Review of Deep-Learning-Based Medical Image Segmentation Methods, Sustainability, 2021, 13(3), 1224."  The second one is not directly related to this domain and was not considered. |

Wherever possible, the changes have been considered in the revised manuscript.

Awaiting for a feedback on the revised version of the paper.

Thank you once again.

Kind Regards,

Maleika Heenaye- Mamode Khan,

On behalf of all authors
